# Supplementary material for: Monoclonal antibody-based immunohistochemistry reveals residual Taenia solium antigens in calcified granulomas from pigs with neurocysticercosis
Source: PLoS Negl Trop Dis. 2026 May 26;20(5):e0014329. doi: 10.1371/journal.pntd.0014329 (PMC13225631; doi:10.1371/journal.pntd.0014329)
Supplement: S1 Table — (DOCX) [file pntd.0014329.s001.docx]

**S1 Table.** Optimized conditions for IHC detection of *T. solium* cyst antigens, including dilutions of primary monoclonal antibodies, blocking agents, and secondary antibodies, validated on brain biopsies with viable brain cysts (positive controls) and uninfected tissue (negative controls for pericystic brain tissue).

| *T. solium* mAb-based IHC assays | Blocking agents | Primary antibody | Secondary antibody |
| --- | --- | --- | --- |
| TsW5 | 0.1% PBS–Triton-X 100 + 0.05% Tween-20 + 6% milk + 10% goat serum | 1/50 | 1/500 |
| TsW8 | 0.1% PBS-Triton-X 100 + 0.05% Tween-20 + 6% milk +  10% goat serum | 1/200 | 1/700 |
| TsW12 | 0.1% PBS-Triton-X 100 + 0.05% Tween-20 + 3% milk +  5% goat serum | 1/5 | 1/500 |
| TsV3 | 0.1% PBS-Triton-X 100 + 0.05% Tween-20 + 6% milk +  5% goat serum | 1/20 | 1/500 |
| TsV4 | 0.1% PBS-Triton-X 100 + 0.05% Tween-20 + 6% milk +  10% goat serum | 1/20 | 1/700 |
| TsE1 | 0.1% PBS-Triton-X 100 + 0.05% Tween-20 + 6% milk +  10% goat serum | 1/50 | 1/700 |
