# Supplementary material for: Monoclonal antibody-based immunohistochemistry reveals residual Taenia solium antigens in calcified granulomas from pigs with neurocysticercosis
Source: PLoS Negl Trop Dis. 2026 May 26;20(5):e0014329. doi: 10.1371/journal.pntd.0014329 (PMC13225631; doi:10.1371/journal.pntd.0014329)
Supplement: S2 Table — (DOCX) [file pntd.0014329.s002.docx]

**S2 Table.** Immunoreactivity areas to *T. solium* cyst antigens (mean % of the total area ± standard errors) as determined by mAb-based IHC assays in calcified granulomas from treated NCC pigs distributed across post-treatment time points.

| Antigen recognition pattern | *T. solium* mAb-based IHC assay | Total  (*N*=20) | Post-treatment time points | | | **P* |
| --- | --- | --- | --- | --- | --- | --- |
|  |  |  | 4 months  (*n*=4) | 8 months  (*n*=10) | 12 months  (*n*=6) |  |
| Antigens trapped within calcified granulomas** | TsW5 | 10.8 ± 3.4 | 21.9 ± 13.2 | 9.3 ± 3.8 | 5.7 ± 3.0 | 0.159 |
|  | TsW8 | 19.4 ± 5.1 | 32.9 ± 12.3 | 17.3 ± 7.4 | 13.8 ± 8.3 | 0.129 |
|  | TsW12 | 14.4 ± 3.5 | 21.1 ± 6.6 | 12.2 ± 5.6 | 13.6 ± 5.9 | 0.386 |
|  | TsV4 | 12.7 ± 3.8 | 23.8 ± 13.2 | 10.8 ± 4.7 | 8.6 ± 5.2 | 0.143 |
|  | TsV3 | 1.5 ± 0.5 | 2.3 ± 2.1 | 1.0 ± 0.4 | 1.9 ± 1.0 | 0.957 |
|  | TsE1 | 1.1 ± 0.4 | 1.5 ± 1.1 | 1.3 ± 0.7 | 0.5 ± 0.3 | 0.498 |
| Antigens present into the perilesional brain parenchyma*** | TsV3 | 4.1 ± 1.2 | 9.0 ± 3.8 | 4.0 ± 1.6 | 0.9 ± 0.4 | 0.010 |
|  | TsE1 | 1.0 ± 0.5 | 4.0 ± 2.2 | 0.3 ± 0.1 | 0.2 ± 0.1 | 0.022 |

**P* values were obtained using the non-parametric Cuzick test for trend

**Immunorreactivity areas were expressed as the percentage of the total granuloma area showing positive staining on IHC

***Immunorreactivity areas were expressed as the percentage of the brain tissue area surrounding calcified granulomas (500 µm) showing positive staining on IHC
