# Supplementary material for: Monoclonal antibody-based immunohistochemistry reveals residual Taenia solium antigens in calcified granulomas from pigs with neurocysticercosis
Source: PLoS Negl Trop Dis. 2026 May 26;20(5):e0014329. doi: 10.1371/journal.pntd.0014329 (PMC13225631; doi:10.1371/journal.pntd.0014329)
Supplement: Attachment — Submitted filename: Itemized_responses_manuscript_antigen_NCC_V2.pdf [file pntd.0014329.s005.pdf]

Itemized responses

Decision on your PLOS Neglected Tropical Diseases submission (PNTD-D-25-02017) - [EMID:148809261f777adb]

Title "Monoclonal antibody-based immunohistochemistry reveals residual Taenia solium antigens in calcified granulomas from pigs with neurocysticercosis"

We thank the Editor and the reviewers for their valuable work and constructive suggestions. We hope that the revised version of the manuscript we are submitting is now acceptable for publication.

JOURNAL REQUIREMENTS

1. Please provide an Author Summary. This should appear in your manuscript between the Abstract (if applicable) and the Introduction and should be 150-200 words long. The aim should be to make your findings accessible to a wide audience that includes both scientists and non-scientists. Sample summaries can be found on our website under Submission Guidelines:
   https://journals.plos.org/plosntds/s/submission-guidelines#loc-parts-of-a-submission

   Response: It has been included as required.

2. We do not publish any copyright or trademark symbols that usually accompany proprietary names, eg. ©, ®, or TM (e.g. next to drug or reagent names). Therefore, please remove all instances of trademark/copyright symbols throughout the text, including:
   - ® on page: 11.
   - TM on page: 11.

   Response: It has been modified as suggested

3. We have noticed that you have uploaded Supporting Information files, but you have not included a list of legends. Please add a full list of legends for your Supporting Information files after the references list.

   Response: It has been modified as suggested.

4. We notice that your supplementary Tables are included in the manuscript file. Please remove them and upload them with the file type 'Supporting Information'. Please ensure that each Supporting Information file has a legend listed in the manuscript after the references list.

   Response: It has been modified as suggested.

REVIEWER COMMENTS

Reviewer's Responses to Questions

Key Review Criteria Required for Acceptance

As you describe the new analyses required for acceptance, please consider the following:

A. METHODS

-Are the objectives of the study clearly articulated with a clear testable hypothesis stated?

-Is the study design appropriate to address the stated objectives?

-Is the population clearly described and appropriate for the hypothesis being tested?

-Is the sample size sufficient to ensure adequate power to address the hypothesis being tested?

-Were correct statistical analysis used to support conclusions?

-Are there concerns about ethical or regulatory requirements being met?

Reviewer #1

This is a very well written manuscript, with a clearly explained objective, methodology and results section. I only have (very) minor comments:

1. How were the 6 mAb selected from the 21 in house anti-*T. solium* mAb? Why these 6?

   <u>Response</u>: It has been added in the revised manuscript (lines 160-162)

2. Did the authors also check for inflammation? Could they have kept sections from each block, eg. the section closest to the section use for IHC?

   <u>Response</u>: We thank the reviewer for this insightful suggestion. It is plausible that the presence of residual parasite antigens may correlate with perilesional tissue damage in calcified granulomas. However, the primary objective of the present study was to demonstrate and characterize patterns of residual antigen recognition using anti-*T. solium* monoclonal antibodies. We agree that further investigations are warranted to co-localize residual parasite antigens with markers of perilesional inflammation, glial activation, gliosis, and neuronal injury to better elucidate the mechanisms underlying seizure development. This point has now been clarified in the Discussion section of the revised manuscript (lines 451–455).

3. Line 104 and 133-134: presence OF…

   <u>Response</u>: It has been corrected (lines 134, and 175).

<u>Reviewer #2</u>

1. The methodology is well described.

   <u>Response</u>: Thanks for your comments

B. RESULTS

- ¿Does the analysis presented match the analysis plan?
- ¿Are the results clearly and completely presented?
- ¿Are the figures (Tables, Images) of sufficient quality for clarity?

Reviewer #1

1. This is a very well written manuscript, with a clearly explained objective, methodology and results section.

   Response: Thanks for your comments

Reviewer #2

1. The results are well described.

   Response: Thanks for your comments.

C. CONCLUSIONS

-Are the conclusions supported by the data presented?

-Are the limitations of analysis clearly described?

-Do the authors discuss how these data can be helpful to advance our understanding of the topic under study?

-Is public health relevance addressed?

Reviewer #1

1. The discussion is also well elaborated, could perhaps be shortened a bit, and includes a relevant limitation section.

   Response: The discussion has been modified. A limitation section has also been updated in the revised manuscript (431-439).

Reviewer #2

1. The conclusion supports the presented data

   Response: thanks for your comments

D. EDITORIAL AND DATA PRESENTATION/MODIFICATION

Use this section for editorial suggestions as well as relatively minor modifications of existing data that would enhance clarity. If the only modifications needed are minor and/or editorial, you may wish to recommend "Minor Revision" or "Accept".

Reviewer #1:

(No Response)

Reviewer #2:

-

E. SUMMARY AND GENERAL COMMENTS

Reviewer #1

This manuscript describes the analyses of calcified cysts collected from NCC pig brains (of treated pigs), using monoclonal antibodies to identify and localize the presence of specific antigens, at different time points. Results may contribute greatly to the understanding of inflammatory processes (and seizures) around calcified cysts in human. This is a very well written manuscript, with a clearly explained objective, methodology and results section. The discussion is also well elaborated, could perhaps be shortened a bit, and includes a relevant limitation section. I only have (very) minor comments:

1. How were the 6 mAb selected from the 21 in house anti-*T. solium* mAb? Why these 6?

Response: Information about selection of six mAbs for IHC is added in the revised manuscript (lines 160-162)

2. Did the authors also check for inflammation? Could they have kept sections from each block, e.g. the section closest to the section use for IHC?

   Response: It has been included in the revised manuscript (lines 355-357, and 451-455).

3. Line 104 and 133-134: presence OF…

   Response: Modified as suggested.

Reviewer #2

The article is well written and discussed, the methodology is acceptable, and both the methodology and results are well described. The study findings may help in understanding the neuropathology of NCC. Some comments need to be addressed by the authors.

1. Why did the authors not test for cross-reactivity using positive samples representing microbial infections?

   Response: We thank the reviewer for raising this point. Assessing the potential cross-reactivity with other microbial infections with our IHC assay is relevant when developing diagnostic immunodetection assays. However, the primary objective of the present study was not to establish a diagnostic tool, but rather to characterize residual parasite antigen recognition patterns within calcified lesions using well-characterized anti-*T. solium* mAbs previously validated for species specificity, with minimal cross-reactivity to non-taeniid pathogens. Additional cross-reactivity to unrelated microbial infections is considered beyond the scope of the mechanistic investigation reported here. We clarify this limitation in the Discussion section of the revised manuscript (lines 436-439).

2. How does the author explain the presence of immunoreactive variabilities among the used antigens?

Response: We thank the reviewer for this important observation. The variability in immunoreactivity among the antigens likely reflects biological heterogeneity in the composition and spatial distribution of residual parasite material within calcified lesions. Calcification is a dynamic process, and parasite-derived components may persist differentially depending on antigen stability, structural localization, degradation kinetics, and epitope accessibility. Differences in monoclonal antibody targets, including variation in antigen abundance and preservation within mineralized tissue, may further contribute to differences in staining intensity and distribution. Importantly, this variability supports the concept of heterogeneous antigen persistence across lesions rather than undermining assay specificity. This interpretation has been clarified in the revised Discussion section (lines 367-378).

3. The author needs to compare (with statistical significance) lesional and peri-lesional concentrations of the antigens at different time points in treated and non-treated NCC pigs to support the hypothesis of release of cyst antigens.

   Response: We appreciate this suggestion. We agree that formally testing antigen release would require quantitative comparisons between lesional and perilesional areas across time points in treated and untreated NCC pigs. Our study was designed to optimize mAb-based IHC and characterize antigen distribution patterns in calcified granulomas, and it did not include a predefined quantitative framework, untreated longitudinal controls, or sufficient sample size to support statistical inference of release. We have revised the manuscript to frame the possibility of antigen release as hypothesis-generating and to clarify that future studies with paired quantification, appropriate controls, and adequate sample size are needed to directly test this mechanism (see lines 431-436).

4. ¿Is this the first evidence of cyst antigen presence and persistence in calcified lesions and surrounding cerebral tissues? If not, state other similar evidence and comment.

   Response: We thank the reviewer for this question. To our knowledge, direct demonstration of persistent parasite antigens within calcified lesions in NCC, and particularly in adjacent brain tissue using species-specific mAbs is limited. Most evidence in the literature has been indirect and based on histopathological description of morphological remnants in biopsies from NCC cases. Our study provides more direct evidence of residual antigen detection, recognition, and patterns of distribution in calcified lesions

5. The authors claim that their findings support the hypothesis that residual parasite antigens persisting in calcified lesions and intermittently released in the perilesional brain tissue may elicit episodic inflammatory responses. However, they did not test for these hypotheses

   Response: We appreciate the reviewer for this important comment. We agree that our study was not designed to directly test the hypothesis that residual antigen release induces episodic inflammatory responses. Our findings demonstrate persistent parasite antigens. While prior imaging and clinical studies have associated calcified lesions with intermittent perilesional inflammation, our data do not establish causality. We now clarify that our conclusions are hypothesis-generating rather than definitive evidence of mechanistic induction of inflammation (lines 355–357).

F. FIGURE RESUBMISSION

While revising your submission, we strongly recommend that you use PLOS's NAAS tool (https://ngplosjournals.pagemajik.ai/artanalysis) to test your figure files. NAAS can convert your figure files to the TIFF file type and meet basic requirements (such as print size, resolution) or provide you with a report on issues that do not meet our requirements and that NAAS cannot fix.

After uploading your figures to PLOS's NAAS tool - https://ngplosjournals.pagemajik.ai/artanalysis, NAAS will process the files provided and display the results in the "Uploaded Files" section of the page as the processing is complete. If the uploaded figures meet our requirements (or NAAS is able to fix the files to meet our requirements), the figure will be marked as "fixed" above. If NAAS is unable to fix the files, a red "failed" label will appear above. When NAAS has confirmed that the figure files meet our requirements, please download the file via the download option, and include these NAAS processed figure files when submitting your revised manuscript.

Reproducibility:

To enhance the reproducibility of your results, we recommend that authors of applicable studies deposit laboratory protocols in protocols.io, where a protocol can be assigned its own identifier (DOI) such that it can be cited independently in the future. Additionally, PLOS ONE offers an option to publish peer-reviewed clinical study protocols. Read more information on sharing protocols

at https://plos.org/protocols?utm_medium=editorial-email&utm_source=authorletters&utm_campaign=protocols
